# Supplementary material for: Overcoming attenuation bias in regressions using polygenic indices
Source: Nat Commun. 2023 Jul 25;14:4473. doi: 10.1038/s41467-023-40069-4 (PMC10368647; doi:10.1038/s41467-023-40069-4)
Supplement: Supplementary file 1 — Supplementary Information [file 41467_2023_40069_MOESM1_ESM.pdf]

Supplementary Information

Overcoming Attenuation Bias in Regressions using Polygenic Indices

Hans van Kippersluis[1,2,*], **Pietro Biroli**[3], **Rita Dias Pereira**[1,2], **Titus J. Galama**[1,2,4,5], **Stephanie von Hinke**[1,2,6], **S. Fleur W. Meddens**[1,7], **Dilnoza Muslimova**[1,2], **Eric A.W. Slob**[1,8,9], **Ronald de Vlaming**[2,4], **and Cornelius A. Rietveld**[1,2,9]

[1]Erasmus School of Economics, Erasmus University Rotterdam, Rotterdam, The Netherlands

[2]Tinbergen Institute, The Netherlands

[3]Department of Economics, University of Bologna, Bologna, Italy

[4]School of Business and Economics, Vrije Universiteit Amsterdam, Amsterdam, The Netherlands

[5]Center for Social and Economic Research, University of Southern California, Los Angeles, United States

[6]School of Economics, University of Bristol, Bristol, United Kingdom

[7]Statistics Netherlands, The Netherlands

[8]Medical Research Council Biostatistics Unit, Cambridge University, Cambridge, United Kingdom

[9]Erasmus University Rotterdam Institute for Behavior and Biology, Rotterdam, The Netherlands

[*]Corresponding author: Erasmus School of Economics, Erasmus University Rotterdam, Burgemeester Oudlaan 50, 3062PA, Rotterdam, The Netherlands, Phone: +31(0)10 4088837, E-mail: hvankippersluis@ese.eur.nl

Contents

1 Supplementary Methods 1: Simulations using GNAMES **3**

1.1 Initialisation . . . 3

1.2 Simulating phenotypes subject to genetic-nurture effects . . . 3

1.3 Assortative mating . . . 4

1.4 Partitioning data . . . 4

1.5 Imperfect genetic correlation . . . 5

1.6 Simulation designs . . . 5

1.7 Meta-analysis as implemented in GNAMES . . . 7

1.8 Correlation between direct and indirect genetic effects . . . 8

1.9 True coefficient when genetic nurture effects are present . . . 9

2 Supplementary Results 1: RMSE **14**

3 Supplementary Results 2: Robustness Simulation Results **17**

3.1 Choice of h^2_{SNP} . . . 17

3.2 Sample size GWAS . . . 18

3.3 No standardisation . . . 19

4 Supplementary Methods 2: Data Empirical Analysis **21**

4.1 Relatedness . . . 21

4.2 Phenotypes . . . 21

4.3 GWAS . . . 21

4.4 Meta-Analysis . . . 22

4.5 Polygenic indices . . . 22

5 Supplementary Results 3: Robustness Empirical Analysis **23**

5.1 Diastolic blood pressure . . . 23

5.2 The between-family and within-family component of the PGI . . . 23

5.3 Comparing ORIV with regular IV . . . 23

Supplementary References **26**

1 Supplementary Methods 1: Simulations using GNAMES

In this section, we describe the outline of our simulations. For this purpose we developed a tool called GNAMES (Genetic Nurture and Assortative Mating Effects Simulator) available on https://github.com/devlaming/gnames. General usage and technical details (in terms of computational efficiency, indexing, etc.) are described in the GitHub repository.

1.1 Initialisation

We start with a population comprising N unrelated founders, for which we observe data on M independent SNPs. Let G_{ij} denote the genotype of founder i at SNP j. G_{ij} is drawn from $\text{Binom}(2, f_j)$, where f_j denotes the frequency of the coded allele for SNP j. Each allele frequency is sampled independently from a $\text{Beta}(0.35, 0.35)$ distribution until it lies in $[0.1, 0.9]$.

We draw an $M \times 1$ vector of direct SNP effects β and an $M \times 1$ vector of parental SNP effects γ (i.e., genetic nurture) as follows:

$$\beta \sim N\left(\mathbf{0}, \frac{h^2}{M}\mathbf{D}\right) \text{ and} \tag{1}$$

$$\gamma \sim N\left(\mathbf{0}, \frac{n^2}{2M}\mathbf{D}\right). \tag{2}$$

where $N(\cdot)$ denotes the Normal distribution, $\mathbf{D}$ denotes a diagonal matrix, for which element j,j equals $(2f_j(1-f_j))^{-1}$, and where h^2 denotes the panmictic SNP-based heritability and n^2 the panmictic proportion of variance accounted for by genetic nurture effects. These SNP effects have the following properties:

- rarer variants have bigger effects than common variants, precisely to such an extent that a common and a rare SNP, on average, explain the same proportion of variation in the outcome, which aligns with SNP-effect assumptions made in, for instance, GREML analyses[1];
- for both the direct SNP effects and the genetic nurture effects, we assume complete polygenicity (i.e., all SNPs affect the outcome);
- genetic nurture and direct SNP effects are independent;
- SNP effects remain constant across generations (relative to each other; the overall scale may change to maintain target heritability).

1.2 Simulating phenotypes subject to genetic-nurture effects

In a given Generation t, comprising N individuals, let $\mathbf{G}$ denote the $N \times M$ matrix of raw genotypes (i.e., count data between zero and two) and let $\mathbf{G}_P$ (resp. $\mathbf{G}_M$) denote the matrix of paternal (maternal) genotypes. The equations for the direct genetic component, the genetic nurture component, and the unique environment component can therefore be written as follows:

$$\mathbf{g}^* = \mathbf{G}\beta, \tag{3}$$

$$\mathbf{n}^* = (\mathbf{G}_P + \mathbf{G}_M)\gamma, \text{ and} \tag{4}$$

$$\varepsilon^* \sim N(\mathbf{0}, \mathbf{I}). \tag{5}$$

For the founders (i.e., Generation 0) we set $\mathbf{n}^* \sim N(\mathbf{0}, \mathbf{I})$. Under random mating, $\mathbf{g}^*$ has a variance equal to h^2 and $\mathbf{n}^*$ a variance equal to n^2—these quantities are not expected to change from one generation to the next. Under assortative mating (AM), we have the same variances in Generation 0. However, in that case, the variance of $\mathbf{g}^*$ and of $\mathbf{n}^*$ are both expected to increase over generations, evolving towards the equilibrium heritability (i.e., $h^2_\infty > h^2$) and the equilibrium contribution for the genetic

nurture component (i.e., $n^2_\infty > n^2$). Therefore, to keep 'contemporary' h^2 and n^2 fixed across generations (in order to have a clear benchmark in our simulations), by default, GNAMES empirically standardises these components to have mean zero and unit variance in each generation, yielding $\mathbf{g}$, $\mathbf{n}$, and ε. Because of this standardisation, it holds that:

$$\mathbf{y} = \mathbf{g}\sqrt{h^2} + \mathbf{n}\sqrt{n^2} + \varepsilon\sqrt{1-h^2-n^2}. \tag{6}$$

GNAMES also has an option to disable the per-generation standardisation. In that case, outcome $\mathbf{y}$ in each generation is generated as:

$$\mathbf{y} = \mathbf{g}^* + \mathbf{n}^* + \varepsilon\sqrt{1-h^2-n^2}. \tag{7}$$

We use this option in Supplementary Information 3.3.

1.3 Assortative mating

In a given generation, we match pairs of individuals based on their value for the phenotype (i.e., we assign pairs of individuals to be mates). We follow the matching procedure as proposed by Border et al.[2]. More specifically, individuals are matched such that their phenotypic correlation equals the desired level ρ_{AM}. This parameter is such that $\rho_{AM} = 0$ corresponds to random mating whereas $\rho_{AM} = 1$ correspond to assortative mating (AM) of such a degree that mates have a perfect phenotypic correlation.

We set up this procedure such that first-degree relatives in a given generation (i.e., siblings) cannot be matched. To this end, we assign Sibling 1 from each nuclear family to Group 1 and Sibling 2 from each nuclear family to Group 2, etc. Next, we apply the matching procedure by Border et al.[2] within each group. The phenotypic correlation between mates then still equals the desired level ρ_{AM}, even across groups (as borne out by our simulations).

Each mating pair has $C = 2$ children. Thus, if Generation t comprises N individuals (assuming N is an even number), there are $N/2$ mating pairs, yielding $CN/2 = 2N/2 = N$ children in total. Hence, in Generation $t+1$ there are also N individuals.

1.4 Partitioning data

We consider $T = 10$ generations. That is, we start at founders ($t = 0$), after which we have 10 generations of matching and mating. Assuming N is an even number, by the end of the simulation we have data on $\frac{1}{2}N$ nuclear families. Per nuclear family, we have data on $C = 2$ siblings.

We partition the sibling data into three parts:

- GWAS Sample 1: Consider N_{GWAS} nuclear families, and per nuclear family consider only one of the two siblings for GWAS 1 (omitting parents).
- GWAS Sample 2: Consider N_{GWAS} nuclear families, and per nuclear family consider only one of the two siblings for GWAS 2 (omitting parents).
- Prediction Sample: Consider $\frac{1}{2}N_{prediction}$ nuclear families, and per nuclear family consider both siblings (omitting parents), yielding a total prediction sample size of $N_{prediction}$ (also assuming $N_{prediction}$ is even).

These three samples are non-overlapping. By partitioning the data as such we can perform between-family GWASs and use the resulting SNP-effect estimates to construct PGIs that we can use for between- and within-family prediction. The sample size of both GWAS 1 and GWAS 2 is equal to N_{GWAS} here. The sample size of the meta-analysis equals $N_{MA} = 2N_{GWAS}$. Finally, the sample size for prediction equals $N_{prediction}$.

1.5 Imperfect genetic correlation

To investigate how imperfect genetic correlations between discovery and prediction samples impact the benchmark (OLS regression on the meta-analysis PGI), ORIV, and the PGI-RC, GNAMES also simulates a secondary trait $\mathbf{y}^*$ according to the following set of equations:

$$\mathbf{y}^* = \begin{cases} \widetilde{\mathbf{g}}\sqrt{h^2} + \widetilde{\mathbf{n}}\sqrt{n^2} + \widetilde{\varepsilon}\sqrt{1-h^2-n^2} & \text{in case of per-generation standardisation, and} \\ \widetilde{\mathbf{g}}^* + \widetilde{\mathbf{n}}^* + \widetilde{\varepsilon}\sqrt{1-h^2-n^2} & \text{in case of no per-generation standardisation, where} \end{cases} \tag{8}$$

$$\widetilde{\mathbf{g}}^* = \mathbf{G}\widetilde{\beta}, \tag{9}$$

$$\widetilde{\mathbf{n}}^* = (\mathbf{G}_P + \mathbf{G}_M)\widetilde{\gamma}, \text{ and} \tag{10}$$

$$\widetilde{\varepsilon}^* \sim N(\mathbf{0}, \mathbf{I}), \tag{11}$$

where $\widetilde{\mathbf{g}}^*$ (resp. $\widetilde{\mathbf{n}}^*$ and $\widetilde{\varepsilon}^*$) is standardised to have mean zero and unit variance, denoted by $\widetilde{\mathbf{g}}$ ($\widetilde{\mathbf{n}}$ and $\widetilde{\varepsilon}$). In these equations,

$$\widetilde{\beta} = \rho_G \beta + \sqrt{1-\rho_G^2}\eta \tag{12}$$

$$\widetilde{\gamma} = \rho_G \gamma + \sqrt{1-\rho_G^2}\zeta, \tag{13}$$

where

$$\eta \sim N\left(\mathbf{0}, \frac{h^2}{M}\mathbf{D}\right) \text{ and} \tag{14}$$

$$\zeta \sim N\left(\mathbf{0}, \frac{n^2}{2M}\mathbf{D}\right). \tag{15}$$

Under these definitions, in GWAS Sample 2 and in the Prediction Sample we may consider $\mathbf{y}$ while in GWAS Sample 1 we consider $\mathbf{y}^*$. In this case, the imperfect genetic correlation exists between one GWAS sample versus the other, while the prediction sample has a perfect genetic correlation with one of the two GWAS samples. Similarly, GWAS Sample 1 and GWAS Sample 2 may both consider $\mathbf{y}$ (i.e., both GWASs and the meta-analysis consider $\mathbf{y}$), while the target trait in the prediction sample is $\mathbf{y}^*$. In this case, the imperfect genetic correlation exists only between prediction samples on the one hand, and GWAS samples on the other.

1.6 Simulation designs

We use various simulation designs to generate the results in the main paper. Below, we present each design with its input parameters. In addition to specifying parameter values, we also provide a link to the code for one run of that particular simulation design, and the corresponding Figure or Table in the paper. In a design where we fix $\rho_G = 1$, this is simply short-hand notation for using $\mathbf{y}$ both in GWAS 1, GWAS 2, and in the prediction sample (i.e., the secondary phenotype, $\mathbf{y}^*$ is ignored altogether in this case).

- $N_{prediction} \in \{1,000; 2,000; 4,000; 8,000; 16,000\}$ (Various prediction sample sizes)
  - For $M = 5,000$, $h^2 = 20\%$, $n^2 = 10\%$, $\rho_{AM} = 0$, $\rho_G = 1$, and $N_{GWAS} = 16,000$: see `https://github.com/devlaming/gnames/blob/main/simulations/design3run1.py`
  - This design is used in Figures 1 and 3. Technically, for Figure 1a we would require a design without genetic nurture, but since the SNP-based heritability is held constant and the Figure refers to a between-family setting, in practice this does not matter.
- $N_{GWAS} \in \{1,000; 2,000; 4,000; 8,000; 16,000\}$ (Various GWAS sample sizes)

- For $M = 5,000$, $h^2 = 25\%$, $n^2 = 0\%$, $\rho_{AM} = 0$, $\rho_G = 1$, and $N_{prediction} = 16,000$:
  see https://github.com/devlaming/gnames/blob/main/simulations/design0run1.py
- For $M = 5,000$, $h^2 = 20\%$, $n^2 = 10\%$, $\rho_{AM} = 0$, $\rho_G = 1$, and $N_{prediction} = 16,000$:
  see https://github.com/devlaming/gnames/blob/main/simulations/design1run1.py
- These designs are used in Figure 2 and 3.

• $N_{GWAS} \in \{2,000; 15,000\}$ and $N_{prediction} \in \{1,000; 4,000; 16,000\}$

- For $M = 5000$, $h^2 = 25\%$, $n^2 = 0\%$, $\rho_{AM} = 0$, $\rho_G = 1$:
  see https://github.com/devlaming/gnames/blob/main/simulations/design10run1.py
- This design is used in Table 1.

• $\rho_{AM} \in \{0, 0.25, 0.5, 0.75, 1.0\}$ (Various levels of AM)

- For $M = 5,000$, $h^2 = 25\%$, $n^2 = 0\%$, $\rho_G = 1$, $N_{GWAS} = 16,000$, and $N_{prediction} = 16,000$:
  see https://github.com/devlaming/gnames/blob/main/simulations/design5run1.py
- For $M = 5,000$, $h^2 = 25\%$, $n^2 = 0\%$, $\rho_G = 1$, $N_{GWAS} = 16,000$, and $N_{prediction} = 16,000$, but without standardisation in each generation:
  see https://github.com/devlaming/gnames/blob/main/simulations/design12run1.py
- For $M = 5,000$, $h^2 = 20\%$, $n^2 = 10\%$, $\rho_G = 1$, $N_{GWAS} = 16,000$, and $N_{prediction} = 16,000$:
  see https://github.com/devlaming/gnames/blob/main/simulations/design2run1.py
- These designs are used in Figure 4 and Supplementary Figure 3.

• Perfect ρ_G between GWAS samples, but $\rho_G \in \{0, 0.25, 0.5, 0.75, 1\}$ for GWAS versus prediction sample

- For $M = 5,000$, $h^2 = 25\%$, $n^2 = 0\%$, $N_{GWAS} = 16,000$, and $N_{prediction} = 16,000$:
  see https://github.com/devlaming/gnames/blob/main/simulations/design7run1.py
- This design is used in Figure 5.

• Perfect ρ_G between GWAS Sample 2 and Prediction Sample, but $\rho_G \in \{0, 0.25, 0.5, 0.75, 1\}$ for GWAS Sample 1 versus GWAS Sample 2

- For $M = 5,000$, $h^2 = 25\%$, $n^2 = 0\%$, $N_{GWAS} = 16,000$, and $N_{prediction} = 16,000$:
  see https://github.com/devlaming/gnames/blob/main/simulations/design8run1.py
- This design is used in Figure 5.

• $N_{GWAS} \in \{3,333; 25,000\}$ and $N_{prediction} \in \{1,000; 4,000; 16000\}$

- For $M = 5,000$, $h^2 = 15\%$, $n^2 = 0\%$, $\rho_{AM} = 0$, $\rho_G = 1$:
  see https://github.com/devlaming/gnames/blob/main/simulations/design9run1.py
- This design is used in Supplementary Table 8.

• $N_{GWAS} \in \{1,000; 7,500\}$ and $N_{prediction} \in \{1,000; 4,000; 16,000\}$

- For $M = 5,000$, $h^2 = 50\%$, $n^2 = 0\%$, $\rho_{AM} = 0$, $\rho_G = 1$:
  see https://github.com/devlaming/gnames/blob/main/simulations/design11run1.py

– This design is used in Supplementary Table 8.

- $\rho_{AM} \in \{0,1\}$ (Two levels of AM with high M and N_{GWAS})
  - For $M = 100{,}000$, $h^2 = 20\%$, $n^2 = 10\%$, $\rho_G = 1$, $N_{GWAS} = 50{,}000$, $N_{prediction} = 16{,}000$: see `https://github.com/devlaming/gnames/blob/main/simulations/design4run1.py`
  - This design is used in Supplementary Figure 2.

1.7 Meta-analysis as implemented in GNAMES

In our simulations using GNAMES, we pool the data used in the two main GWASs, and use this pooled data for a third GWAS, which we refer to as a meta-analysis. Although the latter technically is not a meta-analysis, we here show that this pooled simple GWAS is equivalent to a meta-analysis. As such, we can safely refer to the pooled GWAS as a meta-analysis within the context of our simulations.

We assume there are no control variables to take into account. Moreover, we assume both the SNP and outcome have been standardised to mean zero and unit variance, and we assume a given SNP individually to explain only a negligible proportion of the variation in the outcome. Consider SNP $\mathbf{x}$ and outcome $\mathbf{y}$ (both $N \times 1$ vectors). We split the sample in two chunks with size N_1 and N_2, such that $N = N_1 + N_2$. Let $i = 1,2$ denote an index for the two corresponding subsamples.

The GWAS estimate, for the given SNP, in Subsample $i = 1,2$ can be written as follows:

$$\widehat{\beta}_i = \left(\frac{1}{N_i}\mathbf{x}_i^\top \mathbf{x}_i\right)^{-1}\left(\frac{1}{N_i}\mathbf{x}_i^\top \mathbf{y}_i\right) \tag{16}$$

$$= \frac{1}{N_i}\mathbf{x}_i^\top \mathbf{y}_i. \tag{17}$$

where $\mathbf{x}_i$ (resp. $\mathbf{y}_i$) denotes the SNP (outcome) vector in Subsample i, and where the last equality holds because SNPs have been standardised (i.e., $\frac{1}{N_i}\mathbf{x}_i^\top \mathbf{x}_i = 1$).

Moreover, the GWAS z-score in Subsample i can be written as

$$Z_i = \frac{\widehat{\beta}_i}{\sqrt{\frac{1}{N_i}\left(\frac{1}{N_i}\mathbf{x}_i^\top \mathbf{x}_i\right)^{-1}\sigma_{y_i}^2}}, \tag{18}$$

where $\sigma_{y_i}^2$ is the phenotypic variance in Subsample i not accounted for by the given SNP. As SNPs are standardised, we have that $\frac{1}{N_i}\mathbf{x}_i^\top \mathbf{x}_i = 1$. Moreover, as the SNP explains hardly any variation in y, we have that $\sigma_{y_i}^2 \approx 1$. Therefore, we can approximate the right-hand side in Supplementary Equation 18 as follows:

$$Z_i \approx \frac{1}{\sqrt{N_i}}\mathbf{x}_i^\top \mathbf{y}_i. \tag{19}$$

Moreover, under this standardisation the following approximation of the SNP-effect estimate as function of its z-score also holds:

$$\widehat{\beta}_i \approx \frac{1}{\sqrt{N_i}} Z_i. \tag{20}$$

The sample-size weighted meta-analyses z-score[3] is given by

$$Z = \frac{\sum_i \sqrt{N_i} Z_i}{\sqrt{\sum_i N_i}}, \tag{21}$$

which for the case of two subsamples reduces to

$$Z = \frac{\sqrt{N_1} Z_1 + \sqrt{N_2} Z_2}{\sqrt{N_1 + N_2}}. \tag{22}$$

Using Supplementary Equation 19, the meta-analysis z-score can be approximated as follows:

$$Z \approx \frac{\mathbf{x}_1^\top \mathbf{y}_1 + \mathbf{x}_2^\top \mathbf{y}_2}{\sqrt{N_1 + N_2}}. \tag{23}$$

Moreover, using our approximation for $\widehat{\beta}$ as a function of its z-score in Supplementary Equation 20, we can approximate our meta-analysis SNP-effect estimate as a function of the meta-analysis z-score:

$$\widehat{\beta} \approx \frac{1}{\sqrt{N}} Z, \tag{24}$$

where $N = N_1 + N_2$. Combining Supplementary Equations 23 and 24 yields that the meta-analysis SNP-effect estimate can be approximated as follows:

$$\widehat{\beta}_{MA} \approx \frac{1}{N_1 + N_2} \left(\mathbf{x}_1^\top \mathbf{y}_1 + \mathbf{x}_2^\top \mathbf{y}_2 \right). \tag{25}$$

Now, consider the SNP-effect estimate by applying ordinary least squares (OLS) to pooled data directly (instead of using meta-analysis). This OLS estimate equals:

$$\widehat{\beta}_{pooled} = \left(\frac{1}{N_1 + N_2} \left(\mathbf{x}_1^\top \mathbf{x}_1 + \mathbf{x}_2^\top \mathbf{x}_2 \right) \right)^{-1} \left(\frac{1}{N_1 + N_2} \left(\mathbf{x}_1^\top \mathbf{y}_1 + \mathbf{x}_2^\top \mathbf{y}_2 \right) \right), \tag{26}$$

where $\mathbf{x}_1^\top \mathbf{x}_1 = N_1$ and $\mathbf{x}_2^\top \mathbf{x}_2 = N_2$ by virtue of aforementioned SNP standardisation. Thus, this expression reduces to

$$\widehat{\beta}_{pooled} = \left(\frac{1}{N_1 + N_2} (N_1 + N_2) \right)^{-1} \left(\frac{1}{N_1 + N_2} \left(\mathbf{x}_1^\top \mathbf{y}_1 + \mathbf{x}_2^\top \mathbf{y}_2 \right) \right) \tag{27}$$

$$= \frac{1}{N_1 + N_2} \left(\mathbf{x}_1^\top \mathbf{y}_1 + \mathbf{x}_2^\top \mathbf{y}_2 \right) \tag{28}$$

$$\approx \widehat{\beta}_{MA}. \tag{29}$$

Similarly, we also have that $Z_{pooled} \approx Z_{MA}$. This finding aligns with the work by[4], showing that there are no gains in statistical efficiency by analysing pooled data in a GWAS instead of using meta-analysis.

To illustrate the equivalence, we simulate SNP and outcome data, which we then split into two subsamples. We perform a GWAS in each subsample as well as in the pooled data. Finally, we meta-analyse results from the two subsamples, and compare these to results from the GWAS on pooled data. Supplementary Figure 1 shows pooled GWAS z-scores versus meta-analysis z-scores. The two sets of z-scores are virtually indistinguishable; they have the same scale and an R^2 of 99.99%. Therefore, in the context of GNAMES, we can talk about a pooled GWAS and meta-analysis interchangeably.

1.8 Correlation between direct and indirect genetic effects

In our simulation design with both genetic nurture (GN) and assortative mating (AM) there is an emergent correlation between the direct and indirect component, even if the SNP effects shaping the direct and indirect components are independent. The reason why this emerges is rather subtle. First, consider a case in which there is only AM and no GN. The stronger AM is, the more mates are alike phenotypically. On the level of a single SNP (e.g., with alleles A and T) this translates, ceteris paribus, to

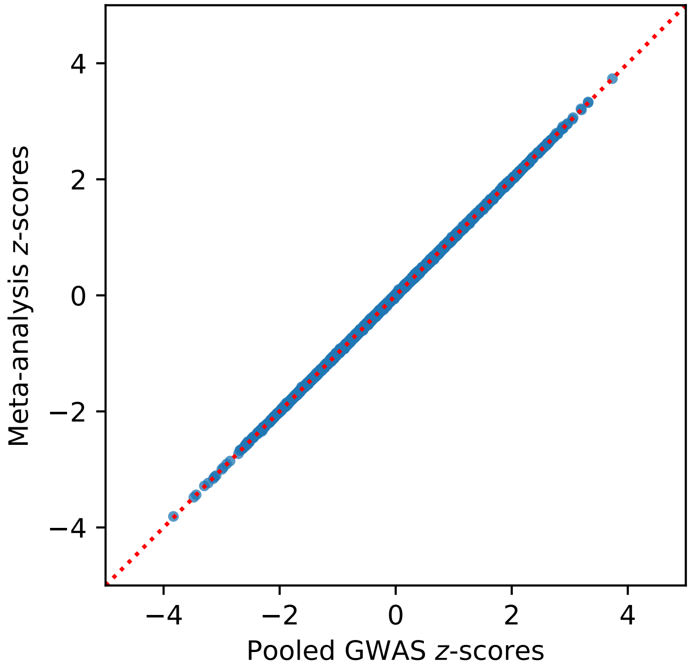

Supplementary Figure 1. Pooled GWAS z-scores versus meta-analysis z-scores ($R^2 = 99.99\%$)

similar genotypes being attracted: an individual with genotype AA mates (more often than randomly) with another individual with genotype AA, and the same goes for TT with TT, and AT with AT.

Now, observe that an AA individual mating with an AA always yields an offspring with the AA genotype (barring mutations). Similarly, TT mating with TT always yields TT offspring. Also, AT individuals mating with AT individuals yield AA offsprings in 25% of the cases, TT also 25%, and AT in 50% of the cases. This illustrates (rather informally) a well-known result: under AM, homozygotes tend to become more prevalent for variants affecting the mating trait. In short, such a SNP will start violating Hardy–Weinberg Equilibrium (HWE) across generations, due to heterozygotes being 'underrepresented' and homozygotes being 'overrepresented'.

Now imagine we have two SNPs, starting in HWE amongst the founders, both affecting the mating trait via direct and indirect effects. When the direct and indirect effects of a given SNP reinforce each other, by having the same sign, such a SNP will tend to violate HWE more quickly than a SNP for which the direct and indirect effect cancel out to some degree, by having a different sign. As the direct and indirect effect are independently drawn, such reinforcement or cancelling out depends purely on chance. However, the SNPs with aligned direct and indirect effects will violate HWE more quickly and, hence, have an inflated variance more rapidly than SNPs for which there is some cancelling out of the direct and indirect effect.

Thus, over generations, the proportion of variance accounted for (VAF) by SNPs with sign-concordant direct and indirect effects (thus affecting the direct and indirect component of the trait in the same direction) is inflated, whereas SNPs with disconcordant direct and indirect effects get deflated, in terms of the proportion of VAF. This pattern, across generations, yields a 'net positive' correlation between the direct and indirect component: the positive part is bolstered, and the negative part diminished.

1.9 True coefficient when genetic nurture effects are present

Here, we derive the expected ORIV coefficient in the absence of assortative mating (AM), but in the presence of genetic nurture (GN), in case the direct SNP effects (δ) and the parental genetic nurture SNP effects (ν) have a non-zero correlation, here denoted by $\rho_{\delta,\nu}$. In this case, the expected coefficient is a function of the correlation $\rho_{\delta,\nu}$. This correlation has a simple

relationship with the correlation between the total direct and indirect component of the trait, denoted by $\rho_{g,n}$. As the combination of genetic nurture (GN) and AM may result in $\rho_{g,n} \neq 0$ even when $\rho_{\delta,\nu} = 0$ (as discussed in Appendix 1.8), we posit that this formula can also be used to give a precise expectation for the ORIV coefficient in that specific scenario. Results from our simulation support our claim that this expectation is indeed accurate.

The data generating process when SNP effects are correlated In the simulation of phenotypes subject to genetic nurture, we followed the simple linear model described in Supplementary Equations 3–6, where the SNP effects were drawn from the distributions outlined in Supplementary Equations 1 and 2. In those equations, scaling of the direct genetic component $\mathbf{g}$ by h^2 and the indirect component $\mathbf{n}$ by n^2 was not done directly via the random SNP effects. Instead, the direct and indirect components were first empirically standardised to mean zero and unit variance, after which they were assigned the appropriate weights in the equation for the phenotype. This process is asymptotically equivalent to formulating y_i (i.e., the phenotype for individual i) as follows:

$$y_i = \sum_{j=1}^{M} (x_{ij}\delta_j + p_{ij}\nu_j + m_{ij}\nu_j) + \varepsilon_i, \text{ where} \tag{30}$$

$$\begin{pmatrix} \delta_j \\ \nu_j \end{pmatrix} \sim N\left(\begin{pmatrix} \mathbf{0} \\ \mathbf{0} \end{pmatrix}, \begin{pmatrix} \frac{h^2}{M} & \frac{1}{M}\rho_{\delta,\nu}\sqrt{\frac{h^2n^2}{2}} \\ \frac{1}{M}\rho_{\delta,\nu}\sqrt{\frac{h^2n^2}{2}} & \frac{n^2}{2M} \end{pmatrix} \right), \tag{31}$$

$$\varepsilon_i \sim N\left(0, 1 - h^2 - n^2\right), \tag{32}$$

where x_{ij} denotes the standardised (instead of raw) SNP data for individual i at SNP j. Similarly, p_{ij} and m_{ij} denote the standardised paternal and maternal SNP data for the same individual at the same SNP. δ_j denotes the direct effect of standardised SNP j, and ν_j the paternal and maternal genetic nurture effect of that standardised SNP. In case $\rho_{\delta,\nu} = 0$, this formulation (i) still ensures the variants jointly explain h^2 in terms of the direct component and n^2 in terms of the indirect component, and (ii) is asymptotically equivalent to the design presented in Supplementary Equations 1–6. From this model, it follows that the phenotypic variance accounted for by SNP j equals:

$$\text{Var}(x_{ij}\delta_j + p_{ij}\nu_j + m_{ij}\nu_j) = \mathbb{E}\left[(x_{ij}\delta_j + p_{ij}\nu_j + m_{ij}\nu_j)^2\right] \tag{33}$$

$$= \mathbb{E}\left[x_{ij}^2\delta_j^2 + p_{ij}^2\nu_j^2 + m_{ij}^2\nu_j^2 + 2x_{ij}p_{ij}\delta_j\nu_j + 2x_{ij}m_{ij}\delta_j\nu_j + 2m_{ij}p_{ij}\nu_j^2\right]. \tag{34}$$

Given SNP effects are uncorrelated with the SNP data, we can rewrite this contribution as:

$$\text{Var}(x_{ij}\delta_j + p_{ij}\nu_j + m_{ij}\nu_j) = \mathbb{E}\left[x_{ij}^2\right]\mathbb{E}\left[\delta_j^2\right] + \mathbb{E}\left[p_{ij}^2\right]\mathbb{E}\left[\nu_j^2\right] + \mathbb{E}\left[m_{ij}^2\right]\mathbb{E}\left[\nu_j^2\right] \tag{35}$$

$$+ 2\mathbb{E}\left[x_{ij}p_{ij}\right]\mathbb{E}\left[\delta_j\nu_j\right] + 2\mathbb{E}\left[x_{ij}m_{ij}\right]\mathbb{E}\left[\delta_j\nu_j\right] + 2\mathbb{E}\left[m_{ij}p_{ij}\right]\mathbb{E}\left[\nu_j^2\right]. \tag{36}$$

Recognising that x_{ij}, p_{ij}, and m_{ij} are mean-centred, the expectations of these random variables (RVs) are equal to the variances (which all equal one, due to standardisation). In addition, we have that $\mathbb{E}\left[\delta_j^2\right] = \text{Var}(\delta_j)$, $\mathbb{E}\left[\nu_j^2\right] = \text{Var}(\nu_j)$, and $\mathbb{E}\left[\delta_j\nu_j\right] = \text{Cov}(\delta_j, \nu_j)$. Moreover, notice that $\mathbb{E}\left[x_{ij}p_{ij}\right]$ is the covariance between the offspring and paternal genotype, which equals the correlation, given standardisation, which equals 0.5. Similarly, $\mathbb{E}\left[x_{ij}m_{ij}\right] = 0.5$. Finally, observe that by the assumption of no AM, we have that $\mathbb{E}\left[m_{ij}p_{ij}\right] = 0$. Thus, the phenotypic variance accounted for (VAF) by SNP j is given by

$$\text{Var}(x_{ij}\delta_j + p_{ij}\nu_j + m_{ij}\nu_j) = \frac{h^2}{M} + \frac{n^2}{M} + \frac{\sqrt{2}}{M}\rho_{\delta,\nu}\sqrt{h^2n^2}. \tag{37}$$

Therefore, aggregating across M SNPs, we have that the phenotypic VAF of all SNPs jointly (in terms of both the direct effect, indirect effect, and their covariance), is given by

$$\mathrm{VAF}_{\mathrm{SNP}} = h^2 + n^2 + \sqrt{2}\rho_{\delta,\nu}\sqrt{h^2n^2}. \tag{38}$$

Relationship between $\rho_{\delta,\nu}$ and $\rho_{g,n}$ Let $\rho_{g,n}$ denote the correlation between the total direct and indirect component for individual i. That is, let

$$g = \sum_{j=1}^{M} x_{ij}\delta_j \text{ and} \tag{39}$$

$$n = \sum_{j=1}^{M} \left(p_{ij}\nu_j + m_{ij}\nu_j\right). \tag{40}$$

As this correlation is the same per SNP, the summation can be ignored without loss of generality. We can now rewrite this correlation as follows:

$$\begin{aligned}
\rho_{g,n} &= \frac{\mathbb{E}\left[x_{ij}\delta_j\left(p_{ij}\nu_j + m_{ij}\nu_j\right)\right]}{\sqrt{\mathbb{E}\left[\left(x_{ij}\delta_j\right)^2\right]\mathbb{E}\left[\left(p_{ij}\nu_j + m_{ij}\nu_j\right)^2\right]}} && (41)\\
&= \frac{\mathbb{E}\left[x_{ij}p_{ij}\delta_j\nu_j + x_{ij}m_{ij}\delta_j\nu_j\right]}{\sqrt{\mathbb{E}\left[x_{ij}^2\delta_j^2\right]\mathbb{E}\left[p_{ij}^2\nu_j^2 + m_{ij}^2\nu_j^2 + 2p_{ij}m_{ij}\nu_j^2\right]}} && (42)\\
&= \frac{0.5\mathbb{E}\left[\delta_j\nu_j\right] + 0.5\mathbb{E}\left[\delta_j\nu_j\right]}{\sqrt{\mathbb{E}\left[\delta_j^2\right]\left(\mathbb{E}\left[\nu_j^2\right] + \mathbb{E}\left[\nu_j^2\right]\right)}} && (43)\\
&= \frac{\frac{1}{M}\rho_{\delta,\nu}\sqrt{\frac{h^2n^2}{2}}}{\sqrt{\frac{h^2}{M}\frac{n^2}{M}}} && (44)\\
&= \frac{\rho_{\delta,\nu}}{\sqrt{2}}. && (45)
\end{aligned}$$

Conversely, we have that $\rho_{\delta,\nu} = \sqrt{2}\rho_{g,n}$. This result shows that the correlation between the direct and indirect component is always smaller than the correlation between the underlying SNP effects in terms of the direct contribution and the contribution via the parental genotypes (i.e., effects δ_j and ν_j). This relationship is intuitive: the total correlation between the direct and indirect component is constrained by two things: imperfect correlation between parent-offspring genotypes (i.e., equal to 0.5 both on the paternal and maternal side) and a given correlation between the SNP effects δ_j and ν_j.

Rescaled h^2 and n^2 when SNP effects are correlated Combining Supplementary Equation 38 and Supplementary Equation 45, the variance accounted for by all SNPs jointly can be written as follows:

$$\mathrm{VAF}_{\mathrm{SNP}} = h^2 + n^2 + 2\rho_{g,n}\sqrt{h^2n^2}. \tag{46}$$

As a result, the phenotypic variance of Y is now given by:

$$\begin{aligned}
\mathrm{Var}\left(y_i\right) &= h^2 + n^2 + 2\rho_{g,n}\sqrt{h^2n^2} + \left(1 - h^2 - n^2\right) && (47)\\
&= 1 + 2\rho_{g,n}\sqrt{h^2n^2}. && (48)
\end{aligned}$$

That is, the variance of Y is different from one when $\rho_{g,n} \neq 0$. This implies that the SNP-based heritability and the proportion of variance accounted for by GN are no longer h^2 and n^2 in this case, but rather:

$$h^2_{adjusted} = \frac{h^2}{1+2\rho_{g,n}\sqrt{h^2 n^2}} \text{ and} \tag{49}$$

$$n^2_{adjusted} = \frac{n^2}{1+2\rho_{g,n}\sqrt{h^2 n^2}}. \tag{50}$$

Only when $\rho_{g,n} = 0$ can h^2 indeed be directly interpreted as the SNP-based heritability (and n^2 interpreted analogously for the nurture component) in our simulations.

Expected coefficient Relying on asymptotic arguments, we can turn our attention to the expected coefficient in case $\rho_{\delta,\nu} = \sqrt{2}\rho_{g,n} \neq 0$. Assuming the data-generating process (DGP) in Supplementary Equation 30 holds true, and writing this DGP in vector notation, we have that

$$\mathbf{y} = \sum_{j=1}^{M} \left(\mathbf{x}_j \delta_j + \mathbf{p}_j \nu_j + \mathbf{m}_j \nu_j \right) + \varepsilon, \tag{51}$$

where bold-faced letters here denote $N \times 1$ vectors. Now, observe that, under aforementioned standardisation of SNP data and the absence of linkage disequilibrium, the GWAS estimator for SNP j in a sample of N unrelated individuals can be written as follows:

$$\widehat{\delta}_j = \left(\frac{1}{N} \mathbf{x}_j^\top \mathbf{x}_j \right)^{-1} \frac{1}{N} \mathbf{x}_j^\top \mathbf{y} \tag{52}$$

$$= \frac{1}{N} \mathbf{x}_j^\top \left(\sum_{k=1}^{M} (\mathbf{x}_k \delta_k + \mathbf{p}_k \nu_k + \mathbf{m}_k \nu_k) + \varepsilon \right) \tag{53}$$

$$= \sum_{k=1}^{M} \left(\frac{1}{N} \mathbf{x}_j^\top \mathbf{x}_k \delta_k + \frac{1}{N} \mathbf{x}_j^\top \mathbf{p}_k \nu_k + \frac{1}{N} \mathbf{x}_j^\top \mathbf{m}_k \nu_k \right) + \frac{1}{N} \mathbf{x}_j^\top \varepsilon \tag{54}$$

$$= \frac{1}{N} \mathbf{x}_j^\top \mathbf{x}_j \delta_j + \frac{1}{N} \mathbf{x}_j^\top \mathbf{p}_j \nu_j + \frac{1}{N} \mathbf{x}_j^\top \mathbf{m}_j \nu_j + \frac{1}{N} \mathbf{x}_j^\top \varepsilon \tag{55}$$

$$= \delta_j + (1+\omega_j)\nu_j + \eta_j, \tag{56}$$

where ω_j is the estimation error in the parent–offspring correlations in genotypes (which is negligible for large N) and where η_j is the GWAS estimation error that ORIV addresses by using a PGI constructed using estimates from one GWAS sample as instrument for a PGI constructed using estimates from another GWAS sample. In other words, for large GWAS sample sizes, ORIV manages to separate the true contribution of $\delta_j + \nu_j$ from the contribution of η_j due to estimation error.

Consider an individual i in the hold-out sample with standardised genotype x_{ij} at SNP j. The contribution of that SNP towards the PGI for that individual that ORIV distills is given by

$$x_{ij}\delta_j + x_{ij}\nu_j. \tag{57}$$

On the other hand, the real contribution of that SNP (also in terms of genetic nurture contributions) towards the phenotype of that individual is given by

$$x_{ij}\delta_j + p_{ij}\nu_j + m_{ij}\nu_j. \tag{58}$$

Hence, we can quantify the phenotypic VAF by ORIV at locus j as follows:

$$\begin{aligned}
\mathrm{Cov}\left(x_{ij}\delta_j + x_{ij}\nu_j, x_{ij}\delta_j + p_{ij}\nu_j + m_{ij}\nu_j\right) &= \mathbb{E}\left[\left(x_{ij}\delta_j + x_{ij}\nu_j\right)\left(x_{ij}\delta_j + p_{ij}\nu_j + m_{ij}\nu_j\right)\right] && (59)\\
&= \mathbb{E}\left[x_{ij}^2\delta_j^2 + x_{ij}p_{ij}\delta_j\nu_j + x_{ij}m_{ij}\delta_j\nu_j + x_{ij}^2\delta_j\nu_j + x_{ij}p_{ij}\nu_j^2 + x_{ij}m_{ij}\nu_j^2\right] && (60)\\
&= \frac{h^2}{M} + 0.5\mathbb{E}\left[\delta_j\nu_j\right] + 0.5\mathbb{E}\left[\delta_j\nu_j\right] + \mathbb{E}\left[\delta_j\nu_j\right] + 0.5\frac{n^2}{2M} + 0.5\frac{n^2}{2M} && (61)\\
&= \frac{h^2}{M} + 2\frac{1}{M}\rho_{\delta,\nu}\sqrt{\frac{h^2n^2}{2}} + \frac{n^2}{2M} && (62)\\
&= \frac{h^2}{M} + \sqrt{2}\frac{1}{M}\rho_{\delta,\nu}\sqrt{h^2n^2} + \frac{n^2}{2M}. && (63)
\end{aligned}$$

Aggregating across all M SNPs, ORIV will, therefore, tag the following part of the phenotypic variance:

$$\begin{aligned}
\mathrm{VAF}_{\mathrm{ORIV}} &= h^2 + \frac{1}{2}n^2 + \sqrt{2}\rho_{\delta,\nu}\sqrt{h^2n^2} && (64)\\
&= h^2 + \frac{1}{2}n^2 + 2\rho_{g,n}\sqrt{h^2n^2}. && (65)
\end{aligned}$$

As a result, the expected coefficient from the true regression under a correlation between the direct and indirect component $\rho_{g,n} \neq 0$ is given by:

$$\text{Expected coefficient} = \sqrt{\frac{h^2 + \frac{1}{2}n^2 + 2\rho_{g,n}\sqrt{h^2n^2}}{1 + 2\rho_{g,n}\sqrt{h^2n^2}}}. \quad (66)$$

Since a positive value for $\rho_{g,n}$ in our simulations arises naturally in a scenario with AM and GN (we did not fix $\rho_{g,n}$ ourselves), we cannot compare our coefficient estimates directly to Supplementary Equation 66 by plugging in the simulated values for h^2, n^2, and $\rho_{g,n}$. Instead, to gauge the performance of ORIV and the PGI-RC in this scenario, we empirically estimate the implied correlation $\hat{\rho}_{g,n}$ from our 100 simulation runs and combine that estimate with our values for h^2 and n^2 to formulate an expected coefficient based on Supplementary Equation 66.

Supplementary Table 1 shows a summary of the simulation results. While the PGI-RC results are biased upwards in the presence of assortative mating, the ORIV estimates are not. When both genetic nurture and assortative mating are present, the target parameter changes due to the arising positive correlation between direct and indirect genetic effects. In this case, ORIV continues to approximate the target parameter very well, whereas the PGI-RC remains biased. These results are graphically presented in Figure 4c in the main text.

Supplementary Table 1. Results accompanying Figure 4c.

AM	$\hat{\rho}_{g,n}$	Expected coefficient	ORIV	PGI-RC
0.00	−0.002	0.500	0.497	0.500
0.25	0.037	0.508	0.506	0.518
0.50	0.080	0.516	0.515	0.536
0.75	0.132	0.526	0.529	0.555
1.00	0.185	0.536	0.538	0.576

Notes: Expected and estimated coefficients for the Polygenic Index (PGI) using Obviously Related Instrumental Variables (ORIV) and the PGI-RC procedure in a scenario with varying levels of assortative mating (AM) and genetic nurture. $\hat{\rho}_{g,n}$ denotes the empirically observed correlation between the direct and indirect genetic component. The simulations hold constant the Genome-wide Association Study (GWAS) discovery sample such that the resulting meta-analysis PGI has an R^2 of 15.4%, and the prediction sample size is held fixed at $N = 16,000$. The simulation results are based on 100 replications.

2 Supplementary Results 1: RMSE

In this section we present the Root Mean Squared Error (RMSE) corresponding to each of the figures in the main text.

Supplementary Table 2. RMSE results accompanying Figure 1.

		Between-family analyses			Within-family analyses	
Prediction sample size	Meta-analysis	ORIV	PGI-RC (Default)	PGI-RC (GREML unc.)	Meta-analysis	ORIV
$N = 1,000$	0.108	0.033	0.124	0.340	0.111	0.061
$N = 2,000$	0.106	0.025	0.088	0.223	0.107	0.039
$N = 4,000$	0.108	0.013	0.054	0.130	0.106	0.028
$N = 8,000$	0.108	0.011	0.028	0.069	0.108	0.027
$N = 16,000$	0.108	0.008	0.018	0.046	0.106	0.018

Notes: Root Mean Squared Error (RMSE) of the estimated coefficients for the Polygenic Index (PGI) using meta-analysis, Obviously Related Instrumental Variables (ORIV), and the PGI-RC procedure (PGI-RC) for varying prediction sample sizes in the baseline scenario (no genetic nurture, no assortative mating) and holding constant the GWAS discovery sample such that the resulting meta-analysis PGI has an R^2 of 15.4%. PGI-RC (Default) refers to the conventional application of the method, whereas PGI-RC (GREML unc.) refers to the case where we incorporate the uncertainty in the estimation of the SNP-based heritability. The simulation results are based on 100 replications.

Supplementary Table 3. RMSE results accompanying Figure 2.

		Between-family analyses			Within-family analyses	
R-squared of meta-analysis PGI	Meta-analysis	ORIV	PGI-RC (Default)	PGI-RC (GREML unc.)	Meta-analysis	ORIV
2.3% ($\sim$ EA1)	0.350	0.075	0.025	0.063	0.346	0.189
4.2% ($\sim$ EA2)	0.295	0.040	0.025	0.061	0.295	0.058
7.1%	0.232	0.020	0.020	0.052	0.229	0.034
11.1% ($\sim$ EA3)	0.166	0.011	0.020	0.050	0.166	0.019
15.4% ($\sim$ EA4)	0.108	0.008	0.017	0.043	0.106	0.018

Notes: Root Mean Squared Error (RMSE) of the estimated coefficients for the Polygenic Index (PGI) using meta-analysis, Obviously Related Instrumental Variables (ORIV), and the PGI-RC procedure (PGI-RC) for varying GWAS discovery sample sizes in the baseline scenario (no genetic nurture, no assortative mating) and holding constant the prediction sample such at $N = 16,000$. PGI-RC (Default) refers to the conventional application of the method, whereas PGI-RC (GREML unc.) refers to the case where we incorporate the uncertainty in the estimation of the SNP-based heritability. The simulation results are based on 100 replications.

Supplementary Table 4. RMSE results accompanying Table 1.

GWAS	Prediction sample	Meta-analysis	ORIV	PGI-RC (Default)	PGI-RC (GREML unc.)
∼EA2	$N = 1,000$	0.291	0.106	0.116	0.334
∼EA2	$N = 4,000$	0.295	0.060	0.072	0.174
∼EA2	$N = 16,000$	0.296	0.035	0.023	0.058
∼EA4	$N = 1,000$	0.108	0.029	0.142	0.387
∼EA4	$N = 4,000$	0.114	0.013	0.046	0.118
∼EA4	$N = 16,000$	0.113	0.008	0.017	0.044

Notes: Root Mean Squared Error (RMSE) of the estimated coefficients for the Polygenic Index (PGI) using meta-analysis, Obviously Related Instrumental Variables (ORIV), and the PGI-RC procedure (PGI-RC) for the baseline scenario (no genetic nurture, no assortative mating), varying both the GWAS discovery sample and prediction sample. PGI-RC (Default) refers to the conventional application of the method, whereas PGI-RC (GREML unc.) refers to the case where we incorporate the uncertainty in the estimation of the SNP-based heritability. Between-family. The simulation results are based on 100 replications.

Supplementary Table 5. RMSE results accompanying Figure 3.

	Between-family analyses				Within-family analyses	
	Meta-analysis	ORIV	PGI-RC (Default)	PGI-RC (GREML unc.)	Meta-analysis	ORIV
Prediction sample size						
$N = 1,000$	0.105	0.029	0.125	0.344	0.129	0.060
$N = 2,000$	0.109	0.024	0.087	0.223	0.130	0.054
$N = 4,000$	0.105	0.016	0.055	0.131	0.131	0.048
$N = 8,000$	0.107	0.012	0.028	0.070	0.133	0.048
$N = 16,000$	0.106	0.007	0.018	0.046	0.132	0.046
R-squared of meta-analysis PGI						
2.3% (∼ EA1)	0.347	0.081	0.021	0.057	0.326	0.187
4.2% (∼ EA2)	0.293	0.039	0.023	0.058	0.283	0.076
7.1%	0.230	0.019	0.022	0.054	0.232	0.049
11.1% (∼ EA3)	0.166	0.011	0.020	0.049	0.179	0.044
15.4% (∼ EA4)	0.106	0.007	0.017	0.043	0.132	0.046

Notes: Root Mean Squared Error (RMSE) of the estimated coefficients for the Polygenic Index (PGI) using meta-analysis, Obviously Related Instrumental Variables (ORIV), and the PGI-RC procedure (PGI-RC) for varying prediction sample sizes in a scenario with genetic nurture but no assortative mating, and holding constant the GWAS discovery sample such that the resulting meta-analysis PGI has an R^2 of 15.4%. PGI-RC (Default) refers to the conventional application of the method, whereas PGI-RC (GREML unc.) refers to the case where we incorporate the uncertainty in the estimation of the SNP-based heritability. The simulation results are based on 100 replications.

Supplementary Table 6. RMSE results accompanying Figure 4.

	Between-family analyses				Within-family analyses	
Assortative mating	Meta-analysis	ORIV	PGI-RC (Default)	PGI-RC (GREML unc.)	Meta-analysis	ORIV
No genetic nurture						
0.00	0.108	0.007	0.016	0.042	0.051	0.057
0.25	0.098	0.008	0.019	0.046	0.052	0.058
0.50	0.089	0.008	0.023	0.052	0.054	0.053
0.75	0.080	0.006	0.029	0.063	0.051	0.056
1.00	0.070	0.006	0.031	0.067	0.050	0.055
Genetic nurture						
0.00	0.109	0.008	0.017	0.044	0.135	0.048
0.25	0.097	0.007	0.018	0.045	0.132	0.049
0.50	0.085	0.007	0.023	0.051	0.129	0.048
0.75	0.069	0.006	0.029	0.061	0.125	0.047
1.00	0.057	0.006	0.040	0.077	0.122	0.050

Notes: Root Mean Squared Error (RMSE) of the estimated coefficients for the Polygenic Index (PGI) using meta-analysis, Obviously Related Instrumental Variables (ORIV), and the PGI-RC procedure (PGI-RC) for varying prediction sample sizes in a scenario with genetic nurture and varying levels of assortative mating, and holding constant the GWAS discovery sample such that the resulting meta-analysis PGI has an R^2 of 15.4%, and the prediction sample at $N = 16,000$. PGI-RC (Default) refers to the conventional application of the method, whereas PGI-RC (GREML unc.) refers to the case where we incorporate the uncertainty in the estimation of the SNP-based heritability. The simulation results are based on 100 replications.

Supplementary Table 7. RMSE results accompanying Figure 5.

	rG GWAS discovery sample – prediction sample				*rG* GWAS discovery samples			
rG	Meta-analysis	ORIV	PGI-RC (Default)	PGI-RC (GREML unc.)	Meta-analysis	ORIV	PGI-RC (Default)	PGI-RC (GREML unc.)
0.25	0.402	0.373	0.078	0.161	0.221	0.132	0.034	0.075
0.50	0.305	0.251	0.049	0.102	0.179	0.029	0.025	0.058
0.75	0.208	0.126	0.026	0.060	0.144	0.011	0.023	0.054
1.00	0.110	0.008	0.023	0.053	0.107	0.009	0.025	0.056

Notes: Root Mean Squared Error (RMSE) of the estimated coefficients for the Polygenic Index (PGI) using meta-analysis, Obviously Related Instrumental Variables (ORIV), and the PGI-RC procedure (PGI-RC) for varying levels of genetic correlation (rG) in the baseline scenario without genetic nurture and no assortative mating, and holding constant the GWAS discovery sample such that the resulting meta-analysis PGI has an R^2 of 15.4% and the discovery sample fixed at $N = 16,000$. PGI-RC (Default) refers to the conventional application of the method, whereas PGI-RC (GREML unc.) refers to the case where we incorporate the uncertainty in the estimation of the SNP-based heritability. The simulation results are based on 100 replications.

3 Supplementary Results 2: Robustness Simulation Results

In this section we describe the Supplementary Results regarding the robustness of our simulation results.

3.1 Choice of h^2_{SNP}

In our main set of simulations we set the SNP-based heritability to 25%, corresponding to $\beta_{st} = 0.5$. Here, we seek to investigate the generalizability of our simulation findings to different levels of SNP-based heritability. In particular, we decrease the SNP-based heritability to 0.15 (i.e., roughly the SNP-based heritability of EA in our empirical analysis) and to 0.5 (i.e., roughly the SNP-based heritability of height in our empirical analysis). In investigating scenarios, for comparability, we maintain the same ratio of the R-squared (R^2) to the SNP-based heritability (h^2_{SNP}) as we use in Table 1. That is, since EA2 roughly corresponds to a ratio of $0.16(= 0.042/0.25)$ and $EA4$ corresponds to a ratio of $0.6(= 0.15/0.25)$, we investigate a similar ratio for the other two levels of heritability. Supplementary Table 8 presents the results.

Supplementary Table 8. Estimated coefficients for the Polygenic Index (PGI) and their corresponding 95% confidence intervals using meta-analysis, Obviously Related Instrumental Variables (ORIV), and the PGI-RC procedure in the baseline scenario (no genetic nurture, no assortative mating; between-family analyses only).

GWAS	Prediction sample	Meta-analysis	ORIV	PGI-RC (Default)	PGI-RC (GREML unc.)
SNP-based heritability = 0.15 ($\beta = 0.387$)					
R^2 / $h^2_{SNP} = 0.16$	$N = 1,000$	0.160	0.435	0.384	0.384
		(0.098 – 0.222)	(-0.150 – 1.021)	(0.231 – 0.537)	(-0.054 – 0.823)
R^2 / $h^2_{SNP} = 0.16$	$N = 4,000$	0.159	0.384	0.379	0.379
		(0.128 –0.190)	(0.229 – 0.538)	(0.304 – 0.453)	(0.191 – 0.566)
R^2 / $h^2_{SNP} = 0.16$	$N = 16,000$	0.158	0.383	0.389	0.389
		(0.142 –0.173)	(0.306 – 0.459)	(0.351 – 0.428)	(0.320 – 0.458)
R^2 / $h^2_{SNP} = 0.6$	$N = 1,000$	0.303	0.392	0.327	0.327
		(0.244 – 0.363)	(0.307 – 0.478)	(0.263 – 0.391)	(-0.101 – 0.756)
R^2 / $h^2_{SNP} = 0.6$	$N = 4,000$	0.299	0.386	0.387	0.387
		(0.269 – 0.329)	(0.344 – 0.429)	(0.349 – 0.426)	(0.233 – 0.541)
R^2 / $h^2_{SNP} = 0.6$	$N = 16,000$	0.300	0.387	0.385	0.385
		(0.285 – 0.315)	(0.366 – 0.408)	(0.366 – 0.405)	(0.331 – 0.440)
SNP-based heritability = 0.5 ($\beta = 0.707$)					
R^2 / $h^2_{SNP} = 0.16$	$N = 1,000$	0.296	0.722	0.682	0.682
		(0.234 – 0.358)	(0.123 – 1.321)	(0.536 – 0.827)	(0.320 – 1.043)
R^2 / $h^2_{SNP} = 0.16$	$N = 4,000$	0.293	0.700	0.705	0.705
		(0.262 – 0.324)	(0.445 – 0.955)	(0.630 – 0.781)	(0.588 – 0.823)
R^2 / $h^2_{SNP} = 0.16$	$N = 16,000$	0.290	0.695	0.707	0.707
		(0.274 – 0.305)	(0.571 – 0.819)	(0.669 – 0.745)	(0.668 – 0.746)
R^2 / $h^2_{SNP} = 0.6$	$N = 1,000$	0.552	0.707	0.660	0.660
		(0.499 – 0.605)	(0.614 – 0.799)	(0.596 – 0.723)	(0.322 – 0.997)
R^2 / $h^2_{SNP} = 0.6$	$N = 4,000$	0.548	0.705	0.709	0.709
		(0.521 – 0.575)	(0.658 – 0.752)	(0.675 – 0.744)	(0.628 – 0.791)
R^2 / $h^2_{SNP} = 0.6$	$N = 16,000$	0.546	0.702	0.707	0.707
		(0.533 – 0.560)	(0.679 – 0.725)	(0.689 – 0.724)	(0.677 – 0.737)

Notes: Coefficients of an OLS regression on a meta-analysis based PGI (column 3), a 2SLS regression using ORIV (column 4), the default PGI-RC (column 5), and the PGI-RC incorporating uncertainty of the GREML estimate (column 6). 95% confidence intervals in parentheses. The simulation results are based on 100 replications.

Similar to Table 1, ORIV is somewhat biased for relatively small GWAS samples, while the PGI-RC is somewhat sensitive to a small prediction sample. Still, both methods clearly outperform a meta-analysis based PGI. For larger GWAS and prediction samples, the two methods both perform accurately, with little differences between them. Applying the default PGI-RC produces slightly narrower confidence intervals. Together, these simulation results show that the performance of a meta-analysis based PGI, ORIV or the PGI-RC are not driven by the absolute value of β_{st} or the SNP-based heritability, such that our conclusions are not specific to any level of SNP-based heritability.

3.2 Sample size GWAS

The expected R-squared of a given PGI in a regression depends on the ratio of the number of SNPs over the GWAS discovery sample. Therefore, to keep the simulation space manageable we proportionally reduced both the number of SNPs as well as the GWAS discovery sample to generate realistic values for the R-squared of the PGIs. In order to verify that this shortcut did not meaningfully affect our results, we conducted a simulation in a scenario with genetic nurture where we increased the number of SNPs from 5,000 to 100,000 and the GWAS discovery sample to 100,000. We hold the discovery sample fixed at $N = 16,000$. In turn, we vary the level of assortative mating ($AM = 0$ or 1).

Supplementary Figure 2 shows a design without assortative mating, and a design with assortative mating of 1, respectively. As can be seen, when holding constant the GWAS discovery sample size, and without assortative mating, Supplementary Figure 2a shows that both ORIV as well as the PGI-RC produce a consistent estimator, whereas a meta-analysis PGI is substantially biased. In case of strong assortative mating, Supplementary Figure 2a again shows that the PGI respository correction is more vulnerable than ORIV in overestimating the true coefficient when the outcome is subject to assortative mating. Supplementary Figure 2b shows that ORIV only slightly underestimates the direct genetic effect within-families irrespective of the level of assortative mating. Overall, the same patterns arise as in our benchmark scenarios, suggesting that the shortcut to proportionally reducing the number of SNPs and the GWAS sample size does not meaningfully affect our conclusions.

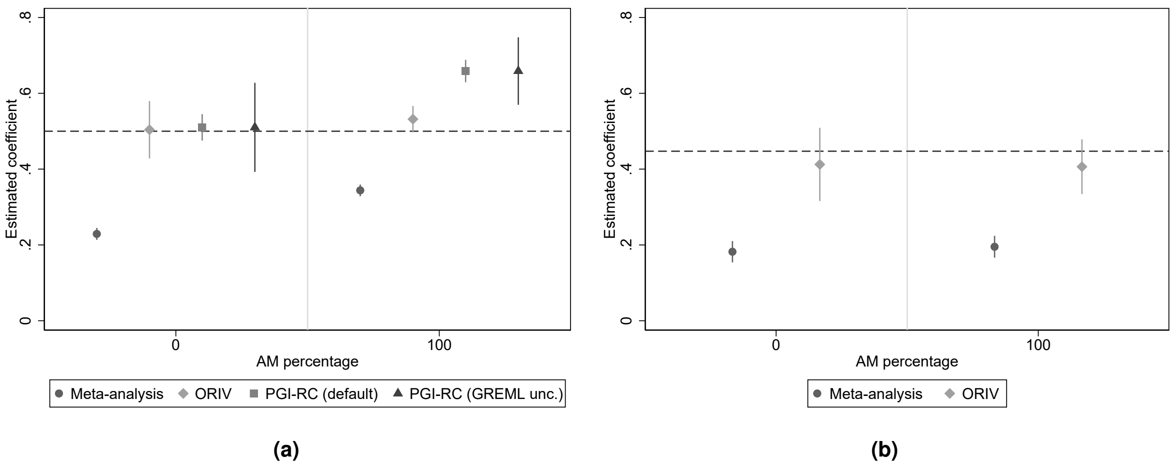

Supplementary Figure 2. Increasing GWAS discovery sample and number of SNPs. a Between-family analyses. **b** Within-family analyses. Data are presented as the estimated coefficients +/- 1.96 times the standard error (95% confidence interval) for the Polygenic Index (PGI) based on between-family analyses (left-panel) and within-family analyses (right-panel) using meta-analysis (circles), Obviously Related Instrumental Variables (ORIV, rhombuses), the default PGI-RC procedure (squares), and the PGI-RC procedure, taking into account uncertainty in the GREML estimates (triangles) in a scenario with genetic nurture, varying assortative mating between 0 and 1, holding constant the prediction sample at $N = 16,000$, and holding constant the GWAS discovery sample at $N = 100,000$ and the number of SNPs also at $M = 100,000$. Dashed line represents the true coefficient. The simulation results are based on 10 replications.

3.3 No standardisation

We designed our baseline simulations such that the true coefficient does not change with increasing levels of assortative mating (AM). To this purpose (as discussed in Supplementary Information 1), we standardise the true PGI to have mean zero and unit variance in each generation of the forward simulation, and then assign effect $\sqrt{h^2}$ to this standardised PGI, when drawing Y as a linear function of this PGI and the other terms in the model. This approach keeps the 'contemporary' heritability fixed (with the exception of the design in which AM and genetic nurture are combined, where the ensuing correlation between the true PGI and nurture component confounds our true coefficient, see Supplementary Information 1.8). Another way to think about our standardisation is that, under AM and the resulting excess homozygosity rates, inflation in the variance of the PGI is avoided by shrinking all SNP effect sizes by the same factor in a given generation. This approach simplifies the formulation of the true coefficient and, hence, gives a clear and intuitive benchmark in all simulation scenarios (again, with the exception of the design in which AM and GN are combined, see Supplementary Information 1.8).

Here, we analyse an alternative implementation without standardisation in each generation. In this scenario, the true coefficient evolves over generations, as the panmictic heritability (i.e., h_0^2 in the notation of Border et al.[2]) evolves towards the equilibrium heritability (i.e., h_∞^2). To the best of our knowledge, the 'contemporary' heritability in a given generation cannot be expressed as a simple function of h_0^2, h_∞^2, and the number of generations passed since the population switched from random mating to AM. Hence, there is no tractable expression available for the true coefficient in that case. Instead, in the simulations without per-generation standardisation in the DGP, we regress the outcome on the true PGI in the prediction sample to obtain an accurate and unbiased estimate of the true between-family coefficient. In addition, to estimate the true within-family coefficient in that scenario, we use the same regression, but then also controlling for family-specific fixed effects.

Supplementary Figure 3 presents the results of these simulations. The true standardized coefficient increases with increasing levels of assortative mating in the absence of standardisation. Still, as in the baseline simulations, ORIV provides consistent estimates of the true coefficient, whereas the PGI-RC overestimates the true coefficient when assortative mating is present.

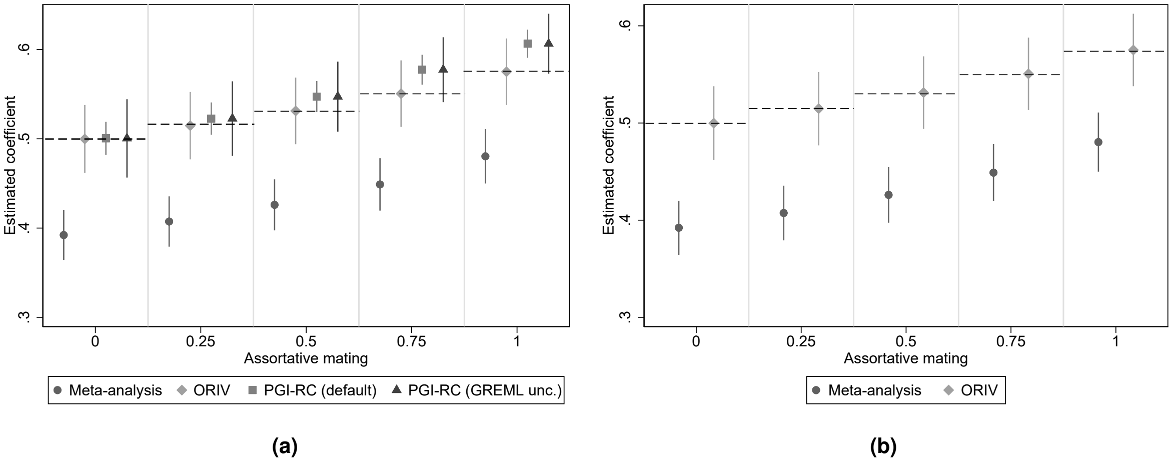

Supplementary Figure 3. Assortative mating without standardisation in each generation. **a** Between-family analyses. **b** Within-family analyses. Data are presented as the estimated coefficients +/- 1.96 times the standard error (95% confidence interval) for the Polygenic Index (PGI) based on between-family analyses (left-panel) and within-family analyses (right-panel) using meta-analysis (circles), Obviously Related Instrumental Variables (ORIV, rhombuses), the default PGI-RC procedure (squares), and the PGI-RC procedure taking into account uncertainty in the GREML estimates (triangles) in a scenario without standardisation of the genetic contributions towards the phenotypes in each generation, without genetic nurture, and with varying levels of assortative mating. The simulations hold constant the Genome-wide Association Study (GWAS) discovery sample such that the resulting meta-analysis PGI has an R^2 of 15.4%, and the prediction sample size is held fixed at $N = 16,000$. The dashed line represents the true coefficient, which increases with higher levels of assortative mating in the absence of standardisation in each generation. The simulation results are based on 100 replications.

4 Supplementary Methods 2: Data Empirical Analysis

The empirical analysis uses siblings in the UK Biobank (UKB)[5] as a prediction sample for constructing the polygenic indices (PGIs). The GWAS discovery sample excludes these full siblings and their relatives up to the 3rd degree to ensure that the discovery and the prediction samples are independent from each other. The analyses were restricted to individuals of European ancestry only. For this purpose, we excluded individuals with a value larger than 0 on the first principal component of the genetic data. We also filtered based on the self-reported ethnicity. Specifically, we only include individuals if their ethnic background is "White", "British", "Irish", or "Any other white background".

4.1 Relatedness

The UKB provides a kinship matrix, which contains the genetically identified degree of relatedness for pairs of UKB participants related up to a third degree or closer. The kinship coefficient is constructed using KING software[6]. We use the following values of the kinship coefficient corresponding to each degree of relatedness: monozygotic twins (> 0.354), first degree parent-child or full siblings (0.177-0.354), and second and third degree relatives and cousins (0.044-0.177). In order to separate parent-child pairs from siblings, we use the identity-by-state (IBS_0) coefficient, which measures genetic similarity in terms of the fraction of markers for which the related individuals do not share alleles[5]. Given their kinship coefficient, parent-child pairs have $IBS_0 < 0.0012$ and full siblings have $IBS_0 > 0.0012$. After classifying all relationships, we separated those who are related to the siblings up to the third degree other than siblings themselves, i.e., parents of siblings and cousins of siblings ($N = 14,947$), and excluded those individuals from the GWAS discovery sample along with the full siblings. This ensures that our prediction sample of siblings is unrelated to the GWAS discovery sample. Importantly, in the GWAS discovery sample there are still some related (non-sibling) individuals. For this reason, we adjust for genetic relatedness in the GWASs. In particular, we kept one random cousin from the cousin clusters before randomly splitting the samples for GWAS.

4.2 Phenotypes

We follow the literature[7–9] and convert an individuals' highest self-reported educational qualification to equivalent years of education using the International Standard Classification of Education (ISCED). The resulting years of education phenotype ranges from 7 to 20, where College or University degree is equivalent to 20 years, National Vocational Qualification (NVQ), Higher National Diploma (HND), or Higher National Certificate (HNC) to 19 years, other professional qualifications to 15 years, having an A or AS levels or similar to 13 years, O levels, and (General) Certificate of Secondary Education ((G)CSE) to 10 years. If "none of the above" is selected, then the lowest level of 7 years is assigned. For height, we use the standing height (in centimeters) of participants measured as a part of the anthropometric data collection at the UKB assessment center. For both phenotypes, we impute the missing values in the first wave with the available information from the two follow up measurements in the UKB.

4.3 GWAS

We perform the GWAS on the UKB discovery sample for educational attainment ($N = 389,419$) and for height ($N = 391,931$), where both exclude siblings and their relatives ($N = 56,450$). For the split-sample PGIs, we first removed all remaining parent-child pairs ($N = 5,084$) and all cousins except one from each cousin cluster ($N = 44,326$). Thereafter, we split the unrelated discovery sample randomly into two parts of $170,004$ for educational attainment and $170,937$ for height. We use the fastGWA approach[10] as implemented in Genome-wide Complex Trait Analysis (GCTA), which applies mixed linear modeling (MLM) to genetic data and requires the following steps. First, based on the relatedness matrix, we generate a sparse genetic relatedness matrix (GRM). We then use the sparse GRM, the SNP data, and the respective phenotype file for height and educational attainment to run the GWAS. Each phenotype file contains a measure of the phenotype residualized with respect to month and year of birth, gender, interaction of birth year and gender, genotyping batch, and the first 40 principal components (PCs) of the genetic relationship matrix. Additional quality control includes cleaning the data with respect to exclusion of

individuals who withdrew consent, with bad genotyping quality, with putative sex chromosome aneuploidy, whose second chromosome karyotypes are different from XX or XY, with outliers in heterozygosity, with missing gender or self-reported gender mismatching with the genetically identified gender, individuals of non-European ancestry, or with missing information on any of the former criteria, on the phenotype or on any of the control variables. Further, we perform quality control on the resulting GWAS summary statistics using the EasyQC tool[11].

4.4 Meta-Analysis

We meta-analyze our own GWAS summary statistics obtained with UKB data with the 23andMe summary statistics for the educational attainment PGI and with the GIANT 2014 summary statistics[12] for the height PGI using the software package METAL[3]. While the GIANT summary statistics are publicly available, the 23andMe summary statistics are only available through 23andMe to qualified researchers under an agreement with 23andMe that protects the privacy of the 23andMe research participants. For more information, visit https://research.23andme.com/collaborate/\#dataset-access/. 23andMe research participants were included in the analysis on the basis of consent status as checked at the time data analyses were initiated. The research participants provided informed consent and participated in the research online, under a protocol approved by the external AAHRPP-accredited IRB, Ethical & Independent Review Services (E&I Review).

4.5 Polygenic indices

We construct the PGIs by accounting for the linkage disequilibrium (LD), i.e., the non-random correlations between SNPs at various loci of a single chromosome, using the LDpred tool[13], version 1.06, and Python, version 3.6.6. LDpred is a Python based package that corrects the GWAS weights for LD using a Bayesian approach. We follow steps as summarized by[14]: (i) coordinate the base and the target files, (ii) compute the LD adjusted weights, and (iii) construct the polygenic index with PLINK[15] using the LD weighted GWAS summary statistics. As LD reference panel, we use 30,000 randomly selected unrelated individuals of EU ancestry in the UKB who passed the quality control protocol. The PGIs are based on approximately 1 million HapMap3 SNPs assuming an infinitesimal model (i.e., we do not apply p-value thresholds). More details can be found in Supplementary Table 9. The final prediction sample consists of $N = 35,282$ siblings.

Supplementary Table 9. Construction details Polygenic Indices.

Trait	GWAS discovery sample	GWAS sample size	#SNPs in PGI
EA	UKB	389,419	1,065,147
	UKB (Split sample 1)	170,004	1,065,147
	UKB (Split sample 2)	170,004	1,065,147
	23andMe	365,536	1,057,144
	UKB + 23andMe	754,955	1,065,061
Height	UKB	391,931	1,065,146
	UKB (Split sample 1)	170,937	1,065,145
	UKB (Split sample 2)	170,937	1,065,145
	GIANT	253,280	969,902
	UKB + GIANT	654,211	1,065,063

5 Supplementary Results 3: Robustness Empirical Analysis

In this section we discuss the robustness of our empirical results.

5.1 Diastolic blood pressure

In Supplementary Table 10, we present the empirical results for Diastolic Blood Pressure (DBP), where we used only a split-sample IV approach on basis of the UKB non-sibling subsample as described in Supplementary Information 4. In line with what can be expected because of the weak assortative mating on this phenotype[16], we do not find strong differences across the between-family (top panel) and within-family (bottom panel) results. Similar as for the other outcomes we studied, a meta-analysis increases the standardized coefficient and increases the predictive power (here, from $\sim 4\%$ to $\sim 6\%$). Applying ORIV further boosts the standardized effect by another 27%. The estimate obtained by the PGI-RC method is even higher, yet is surrounded by larger confidence intervals, especially when taking into the uncertainty of the GREML estimate. Within-families, ORIV again substantially boosts the estimated coefficient by 27%, clearly outperforming an OLS regression on basis of the meta-analysis PGI.

5.2 The between-family and within-family component of the PGI

The correlation between the independent PGIs reflects the measurement error in the PGI of the between-family component. There will be some bias in the within-family estimates if the measurement error of the within-family signal is considerably different from the measurement error of the between-family signal. We therefore computed the correlation between deviations of the PGI from the family means in the UKB, and use this as the scaling factor in the within-family analysis (we thank Elliot Tucker-Drob for this suggestion). The results were very similar compared to the main results. For EA, the within-family two-sample and split-sample coefficients were estimated as 0.179 (0.012) and 0.161 (0.012), respectively. For comparison, the coefficients are 0.184 (0.012) and 0.170 (0.013) in Table 2. For height, the within-family two-sample and split-sample ORIV coefficients were estimated as 0.604 (0.008) and 0.556 (0.009), respectively. In Table 3, the coefficients are 0.614 (0.008) and 0.571 (0.009). Full results for this robustness check are available upon request from the authors. These findings suggest that the measurement error across between- and within-family signals is comparable and does not represent a relevant threat to the application of ORIV for these traits.

5.3 Comparing ORIV with regular IV

In this section we compare the ORIV estimator to the regular IV estimator suggested by DiPrete et al.[17] for our between-family empirical analyses regarding EA (Supplementary Table 11) and height (Supplementary Table 12). To obtain the regular IV estimator, a researcher needs to select which PGI to use as instrumental variable. The Obviously-Related Instrumental Variables (ORIV) approach[18] overcomes the arbitrary choice of selecting one PGI as the independent variable and the other PGI as IV.

The results show that when using summary statistics from two different sources (UKB and 23andMe in the case of EA; UKB and GIANT in the case of height) the IV estimates can vary quite strongly and can be significantly different from each other. In these cases, rather than making an arbitrary choice for one or the other, it seems more appropriate to apply ORIV, where all information is used. In case of the split-sample PGIs, the ORIV and IV point estimates do not diverge as much. Still, even here, ORIV would be recommended as the precision of these estimates is slightly better.

Supplementary Table 10. Results of the OLS and IV regressions explaining (residualized and standardized) Diastolic Blood Pressure (DBP).

	OLS (UKB 1)	OLS (UKB 2)	OLS (Meta-analysis)	ORIV (Split-sample)	PGI-RC (Default)	PGI-RC (GREML unc.)
**Between-family results**						
Polygenic index	0.209***	0.204***	0.249***	0.318***	0.366***	0.366***
	(0.006)	(0.006)	(0.006)	(0.006)	(0.008)	(0.029)
First-stage estimate				0.422***		
				(0.005)		
First-stage F-statistic				6541.31		
Incremental R^2	4.3%	4.2%	6.2%			
Family fixed effects	NO	NO	NO	NO	NO	NO
N	31,977	31,977	31,977	31,977	31,977	31,977
**Within-family results**						
Polygenic index	0.189***	0.204***	0.243***	0.308***		
	(0.010)	(0.010)	(0.010)	(0.014)		
First-stage estimate				0.415***		
				(0.006)		
First-stage F-statistic				3936.66		
Incremental R^2	3.6%	4.1%	5.9%			
Family fixed effects	YES	YES	YES	YES		
N	31,977	31,977	31,977	31,977		

Notes: * p-value < 0.10; ** p-value < 0.05; *** p-value < 0.01 of a two-sided t-test (OLS, PGI-RC) or two-sided z-test (ORIV) without adjustments for multiple comparisons. In all regressions the dependent variable is residualized diastolic blood pressure (standardized to have mean 0 and standard deviation 1), where the residuals are obtained after a regression of diastolic blood pressure controlling for sex, year of birth, month of birth, sex interacted with year of birth, and the first 40 principal components of the genetic relationship matrix. Standard errors are robust and clustered at the family level, and in case of ORIV also at the individual level. OLS (UKB 1/2) refers to models with the PGI constructed using the split-sample UKB non-sibling (i.e., excluding all siblings and their relatives) sample. OLS (Meta-analysis) uses a PGI constructed using a meta-analysis of the two split-sample UKB non-sibling samples. ORIV (Split-sample) refers to a 2SLS estimation where the summary statistics derive from a random split of the UKB sample. PGI-RC refers to the PGI-RC method, where (Default) refers to the conventional application of the method, whereas (GREML unc.) refers to the case where we incorporate the uncertainty in the estimation of the SNP-based heritability.

Supplementary Table 11. Results of the IV regressions explaining (residualized and standardized) educational attainment.

	IV (UKB)	IV (23andMe)	ORIV (2-sample)	IV (UKB 1)	IV (UKB 2)	ORIV (Split-sample)
Polygenic index	0.309***	0.366***	0.337***	0.322***	0.325***	0.323***
	(0.008)	(0.008)	(0.007)	(0.008)	(0.008)	(0.007)
N	35,282	35,282	35,282	35,282	35,282	35,282

Notes: * p-value < 0.10; ** p-value < 0.05; *** p-value < 0.01 of a two-sided z-test without adjustments for multiple comparisons. In all regressions the dependent variable is residualized EA (standardized to have mean 0 and standard deviation 1), where the residuals are obtained after a regression of height on controlling for sex, year of birth, month of birth, sex interacted with year of birth, and the first 40 principal components of the genetic relationship matrix. Family fixed effects are not included. Standard errors are robust and clustered at the family level, and in case of ORIV also at the individual level. IV (UKB) refers to a 2SLS estimation using PGI on the basis of the UKB instrumented by the PGI constructed using the 23andMe summary statistics, while IV (23andMe) is a 2SLS regression where the roles are reversed. ORIV (2-sample) refers to a 2SLS estimation using the PGIs from the UKB non-sibling sample and Giant as instrumental variables for each other. IV (UKB1(2)) refers to a 2SLS estimation using the split-sample UKB PGI 1(2) is used as IV for UKB PGI 2(1). ORIV (Split-sample) refers to a 2SLS estimation where the summary statistics derive from a random split of the UKB sample.

Supplementary Table 12. Results of the IV regressions explaining (residualized and standardized) height.

	IV (UKB)	IV (GIANT)	ORIV (2-sample)	IV (UKB 1)	IV (UKB 2)	ORIV (Split-sample)
Polygenic index	0.571***	0.734***	0.652***	0.625***	0.634***	0.617***
	(0.006)	(0.008)	(0.006)	(0.007)	(0.007)	(0.007)
N	35,282	35,282	35,282	35,282	35,282	35,282

Notes: * p-value < 0.10; ** p-value < 0.05; *** p-value < 0.01 of a two-sided z-test without adjustments for multiple comparisons. In all regressions the dependent variable is residualized height (standardized to have mean 0 and standard deviation 1), where the residuals are obtained after a regression of height on controlling for sex, year of birth, month of birth, sex interacted with year of birth, and the first 40 principal components of the genetic relationship matrix. Family fixed effects are not included. Standard errors are robust and clustered at the family level, and in case of ORIV also at the individual level. IV (UKB) refers to a 2SLS estimation using PGI on the basis of the UKB instrumented by the PGI constructed using the GIANT summary statistics, while IV (GIANT) is a 2SLS regression where the roles are reversed. ORIV (2-sample) refers to a 2SLS estimation using the PGIs from the UKB non-sibling sample and GIANT as instrumental variables for each other. IV (UKB1(2)) refers to a 2SLS estimation using the split-sample UKB PGI 1(2) is used as IV for UKB PGI 2(1). ORIV (Split-sample) refers to a 2SLS estimation where the summary statistics derive from a random split of the UKB sample.

Supplementary References

1. Yang, J. *et al.* Common SNPs explain a large proportion of the heritability for human height. *Nat. Genet.* **42**, 565–9, DOI: 10.1038/ng.608 (2010).
2. Border, R. *et al.* Assortative mating biases marker-based heritability estimators. *Nat. Commun.* **13**, 660, DOI: 10.1038/s41467-022-28294-9 (2022).
3. Willer, C. J., Li, Y. & Abecasis, G. R. Metal: Fast and efficient meta-analysis of genomewide association scans. *Bioinformatics* **26**, 2190–2191, DOI: 10.1093/bioinformatics/btq340 (2010).
4. Lin, D. & Zeng, D. Meta-analysis of genome-wide association studies: No efficiency gain in using individual participant data. *Genet. Epidemiol.* **34**, 60–66, DOI: 10.1002/gepi.20435 (2010).
5. Bycroft, C. *et al.* The UK Biobank resource with deep phenotyping and genomic data. *Nature* **562**, 203–209, DOI: 10.1038/s41586-018-0579-z (2018).
6. Manichaikul, A. *et al.* Robust relationship inference in genome-wide association studies. *Bioinformatics* **26**, 2867–2873, DOI: 10.1093/bioinformatics/btq559 (2010).
7. Lee, J. J. *et al.* Gene discovery and polygenic prediction from a 1.1-million-person GWAS of educational attainment. *Nat. Genet.* **50**, 1112–1121, DOI: 10.1038/s41588-018-0147-3 (2018).
8. Okbay, A. *et al.* Genome-wide association study identifies 74 loci associated with educational attainment. *Nature* **533**, 539–542, DOI: 10.1038/nature17671 (2016).
9. Rietveld, C. A. *et al.* GWAS of 126,559 individuals identifies genetic variants associated with educational attainment. *Science* **340**, 1467–1471, DOI: 10.1257/jep.25.4.57 (2013).
10. Jiang, L. *et al.* A resource-efficient tool for mixed model association analysis of large-scale data. *Nat. Genet.* **51**, 1749–1755, DOI: 10.1038/s41588-019-0530-8 (2019).
11. Winkler, T. W. *et al.* Quality control and conduct of genome-wide association meta-analyses. *Nat. Protoc.* **9**, 1192–1212, DOI: 10.1038/nprot.2014.071 (2014).
12. Wood, A. R. *et al.* Defining the role of common variation in the genomic and biological architecture of adult human height. *Nat. Genet.* **46**, 1173–1186, DOI: 10.1038/ng.3097 (2014).
13. Vilhjalmsson, B. J. *et al.* Modeling linkage disequilibrium increases accuracy of polygenic risk scores. *Am. J. Hum. Genet.* **97**, 576–592, DOI: 10.1016/j.ajhg.2015.09.001 (2015).
14. Mills, M., Barban, N. & Tropf, F. C. *An Introduction to Statistical Genetic Data Analysis* (MIT Press, 2020).
15. Chang, C. C. *et al.* Second-generation PLINK: Rising to the challenge of larger and richer datasets. *Gigascience* **4**, S13742–015, DOI: 10.1186/s13742-015-0047-8 (2015).
16. Robinson, M. R. *et al.* Genetic evidence of assortative mating in humans. *Nat. Hum. Behav.* **1**, 0016, DOI: 10.1038/s41562-016-0016 (2017).
17. DiPrete, T. A., Burik, C. A. P. & Koellinger, P. D. Genetic instrumental variable regression: Explaining socioeconomic and health outcomes in nonexperimental data. *Proc. Natl. Acad. Sci.* **115**, E4970–E4979, DOI: 10.1073/pnas.1707388115 (2018).
18. Gillen, B., Snowberg, E. & Yariv, L. Experimenting with measurement error: Techniques with applications to the Caltech cohort study. *J. Polit. Econ.* **127**, 1826–1863, DOI: 10.1086/701681 (2019).
