# Supplementary material for: Overcoming attenuation bias in regressions using polygenic indices
Source: Nat Commun. 2023 Jul 25;14:4473. doi: 10.1038/s41467-023-40069-4 (PMC10368647; doi:10.1038/s41467-023-40069-4)
Supplement: Supplementary file 3 — Reporting Summary [file 41467_2023_40069_MOESM3_ESM.pdf]

nature portfolio

Corresponding author(s): Hans van Kippersluis

Last updated by author(s): Jun 14, 2023

Reporting Summary

Nature Portfolio wishes to improve the reproducibility of the work that we publish. This form provides structure for consistency and transparency in reporting. For further information on Nature Portfolio policies, see our Editorial Policies and the Editorial Policy Checklist.

Statistics

For all statistical analyses, confirm that the following items are present in the figure legend, table legend, main text, or Methods section.

n/a	Confirmed	
☐	☒	The exact sample size (*n*) for each experimental group/condition, given as a discrete number and unit of measurement
☐	☒	A statement on whether measurements were taken from distinct samples or whether the same sample was measured repeatedly
☐	☒	The statistical test(s) used AND whether they are one- or two-sided<br>*Only common tests should be described solely by name; describe more complex techniques in the Methods section.*
☐	☒	A description of all covariates tested
☐	☒	A description of any assumptions or corrections, such as tests of normality and adjustment for multiple comparisons
☐	☒	A full description of the statistical parameters including central tendency (e.g. means) or other basic estimates (e.g. regression coefficient) AND variation (e.g. standard deviation) or associated estimates of uncertainty (e.g. confidence intervals)
☐	☒	For null hypothesis testing, the test statistic (e.g. *F*, *t*, *r*) with confidence intervals, effect sizes, degrees of freedom and *P* value noted<br>*Give P values as exact values whenever suitable.*
☒	☐	For Bayesian analysis, information on the choice of priors and Markov chain Monte Carlo settings
☒	☐	For hierarchical and complex designs, identification of the appropriate level for tests and full reporting of outcomes
☒	☐	Estimates of effect sizes (e.g. Cohen's *d*, Pearson's *r*), indicating how they were calculated

Our web collection on statistics for biologists contains articles on many of the points above.

Software and code

Policy information about availability of computer code

Data collection	All syntax and details on the simulation analyses are available on https://github.com/devlaming/gnames.
Data analysis	All syntax for the empirical analyses conducted in Stata version 17 and PLINK version 1.9 can be found on https://github.com/geighei/ORIV.

For manuscripts utilizing custom algorithms or software that are central to the research but not yet described in published literature, software must be made available to editors and reviewers. We strongly encourage code deposition in a community repository (e.g. GitHub). See the Nature Portfolio guidelines for submitting code & software for further information.

Data

Policy information about availability of data

All manuscripts must include a data availability statement. This statement should provide the following information, where applicable:

- Accession codes, unique identifiers, or web links for publicly available datasets
- A description of any restrictions on data availability
- For clinical datasets or third party data, please ensure that the statement adheres to our policy

This research has been conducted using the UK Biobank resource (Application number 41382). UK Biobank data can be applied for through \url{https://www.ukbiobank.ac.uk/enable-your-research/apply-for-access}.

Research involving human participants, their data, or biological material

Policy information about studies with human participants or human data. See also policy information about sex, gender (identity/presentation), and sexual orientation and race, ethnicity and racism.

Reporting on sex and gender	We use the term sex as a biological attribute as a confounder in our empirical analysis. We do not stratify the sample by sex as it is mainly a methodological paper, where further stratification into subgroups would distract the reader from the main contribution of the paper.
Reporting on race, ethnicity, or other socially relevant groupings	We adjust for sex, year of birth, month of birth, sex interacted with year of birth, and the first 40 principal components of the genetic relationship matrix. In line with the literature, we restrict to European-ancestry individuals in the UK Biobank and so controls for ethnicity are not needed.
Population characteristics	See above.
Recruitment	We use the UK Biobank, which is a self-selected sample and we reflect on this in the limitations section.
Ethics oversight	No ethical approval was obtained for this methodological study on the basis of secondary data.

Note that full information on the approval of the study protocol must also be provided in the manuscript.

Field-specific reporting

Please select the one below that is the best fit for your research. If you are not sure, read the appropriate sections before making your selection.

☐ Life sciences ☒ Behavioural & social sciences ☐ Ecological, evolutionary & environmental sciences

For a reference copy of the document with all sections, see nature.com/documents/nr-reporting-summary-flat.pdf

Behavioural & social sciences study design

All studies must disclose on these points even when the disclosure is negative.

Study description	We rely on simulated and quantitative empirical data from the UK Biobank to estimate Ordinary Least Squares (OLS) and Instrumental Variables (IV) regressions of an outcome (educational attainment, height and blood pressure) on their respective polygenic indices. The goal is to compare the attenuation bias as well as the precision of the regression coefficients of three methods (OLS, ORIV and the Polygenic Index Repository Correction) under various scenario's.
Research sample	We rely on the existing dataset of the UK Biobank. In our empirical analyses, we restrict to the sibling subsample from European ancestry (N=35,282). The reason for restricting to this subsample is twofold: (i) it enables to estimate our own tailor-made GWAS on a large remaining part of the UK Biobank (the non-siblings); and (ii) restricting to siblings facilitates running between- as well as within-family analyses. The respondents are born between 1937 and 1970, live in the United Kingdom and around 43% of the sample is male.
Sampling strategy	The sampling strategy for the UK Biobank itself was by post mail letters send out to over 5 million UK residents. The response rate was around 10% and tends to somewhat selective on education and health outcomes. In our analysis, we use all siblings in the UK Biobank data with full information on the relevant variables were selected and that passed quality control on the genetic information. Given the known predictive power of the polygenic indices, no sample size calculations were performed.
Data collection	We rely on secondary data from the UK Biobank. See https://www.ukbiobank.ac.uk/ and above for details on their data collection.
Timing	The UK Biobank data was collected initially between 2006 and 2010
Data exclusions	From the original UK Biobank sample, drop 201 individuals who withdrew consent in the mean time, we drop 92,893 non-European ancestry individuals, we drop 374,122 individuals who did not share any siblings, and 3602 for whom one of our main variables was missing. These criteria were pre-established and standard albeit not formally written down in a pre-analysis plan.
Non-participation	No participants were involved in the study.
Randomization	We rely on secondary data so randomization was not possible in this study.

Reporting for specific materials, systems and methods

We require information from authors about some types of materials, experimental systems and methods used in many studies. Here, indicate whether each material, system or method listed is relevant to your study. If you are not sure if a list item applies to your research, read the appropriate section before selecting a response.

Materials & experimental systems

n/a	Involved in the study
☒	☐ Antibodies
☒	☐ Eukaryotic cell lines
☒	☐ Palaeontology and archaeology
☒	☐ Animals and other organisms
☒	☐ Clinical data
☒	☐ Dual use research of concern
☒	☐ Plants

Methods

n/a	Involved in the study
☒	☐ ChIP-seq
☒	☐ Flow cytometry
☒	☐ MRI-based neuroimaging
